# Fréquence et profil clinique de la maladie de Parkinson et des autres syndromes Parkinsoniens vus au service de neurologie de l'hôpital Befelatanana Antananarivo

**DOI:** 10.11604/pamj.2019.33.229.19361

**Published:** 2019-07-18

**Authors:** Nomena Finiavana Rasaholiarison, Julien Razafimahefa, Jenny Larissa Rakotomanana, Alain Djacoba Tehindrazanarivelo

**Affiliations:** 1 Service de Neurologie, Hôpital Befelatanana, Antananarivo, Madagascar

**Keywords:** Madagascar, maladie de Parkinson, syndrome parkinsonien atypique, syndrome parkinsonien secondaire, Madagascar, Parkinson's disease, atypical parkinsonian syndrome, secondary parkinsonian syndrome

## Abstract

**Introduction:**

Actuellement nous n'avons pas de données précises sur les syndromes parkinsoniens à Madagascar. Nous voulions rassembler des données sur ces maladies. Alors l'objectif de notre étude était de décrire la fréquence et le profil clinique des syndromes parkinsoniens dans notre Service de Neurologie.

**Méthodes:**

Il s'agit d'une étude rétrospective descriptive allant de janvier 2014 à juin 2018 dans le Service de Neurologie, Befelatanana. Les données démographiques et cliniques des patients diagnostiqués comme ayant un syndrome parkinsonien étaient collectées. Nous en avons évalué les fréquences et les caractéristiques puis comparé les patients avec la Maladie de Parkinson idiopathique et les autres syndromes parkinsoniens. Les données étaient traitées par le logiciel R.

**Résultats:**

Nous avons retenus 104 patients sur 3528, vus dans notre service. Parmi les patients avec un syndrome parkinsonien, 67 (64,42%) avaient une maladie de Parkinson idiopathique (MP) et 37 (35,47%) un syndrome parkinsonien autre. L'intervalle moyen entre le début de la maladie et la consultation ou l'hospitalisation dans le service était de 2,5 ans. Pour les MP, l'âge moyen de début était à 58,5 [23; 80] ans, l'âge du diagnostic à 62 [28; 83] ans et la sex-ratio était de 1,97. La médiane de score de Hoehn et Yahr était de 2.0. Les formes de MP étaient tremblantes dans 24 (35,42%), mixte 28 (41,71%) et akineto-rigide 15 (22,38%) des cas. Pour les autres syndromes parkinsoniens, 27 (72,97%) étaient des hommes, l'âge moyen de début était à 57,5 [26; 83] ans, l'âge du diagnostic à 59,3 [26; 83] ans, les étiologies étaient dominées par l'atrophie multi-systématisée avec 17/37 (46,64%). Les patients atteints d'autres syndromes parkinsoniens avaient plus de troubles cognitifs (p=0,0306) et les MP étaient plus sensible au DOPAMINE (p=0,006).

**Conclusion:**

Les patients parkinsoniens idiopathiques avaient des caractéristiques différentes de ceux avec autres parkinsonismes. Il y a avait eu un retard diagnostique comme dans les autres pays en voie de développement.

## Abstract (en)

**Introduction:**

Currently we have no precise data on the parkinsonian syndromes in Madagascar. This study aims to collect data on these diseases and to describe the frequency and the clinical profile of parkinsonian syndromes in our Department of Neurology.

**Methods:**

We conducted a retrospective and descriptive study in the Department of Neurology, in Befelatanana from January 2014 to June 2018. The demographic and clinical data of patients diagnosed as having a parkinsonian syndrome were collected. We assessed data rates and characteristics and then we compared patients with idiopathic Parkinson's disease and patients with other parkinsonian syndromes. Data were processed using R. software.

**Results:**

The study included 104 of 3528 patients, seen in our Department. Among the patients with parkinsonian syndrome, 67(64.42%) had idiopathic Parkinson's disease (PD) and 37 (35.47%) another parkinsonian syndrome. The average interval between the onset of the disease and the consultation or the hospitalization in the Department was 2.5 years. For MP, the median age at onset was 58.5 [23; 80] years, the median age at diagnosis was 62 [28; 83] years and the sex ratio were 1.97. The median Hoehn and Yahr score were 2.0. Tremor-dominant Parkinson's disease was found in 24 (35.42%) of cases, a mixed phenotype was found in 28 (41.71%) of cases while akineto-rigid Parkinson's disease was found in 15 (22.38%) of cases. The other parkinsonian syndromes ocuurred in 27 (72.97%) men, with a median age at onset of 57.5 [26; 83] years, a median age at diagnosis of 59.3 [26; 83] years. Etiologies were dominated by Multiple System Atrophy 17/37 (46.64%). Patients with other parkinsonian syndromes had several cognitive disorders (p=0,0306) and MPs were more sensitive to dopamine (p=0.006).

**Conclusion:**

The patients with idiopathic parkinsonian syndrome had features different from those of patients with parkinsonism. There was a diagnostic delay as in other developing countries.

## Introduction

Le syndrome parkinsonien est un ensemble de troubles moteurs qui partage les caractéristiques suivant: tremblements, bradykinésie et rigidité [1]. Ils peuvent être classés en syndrome parkinsonien dégénérative ou primaire regroupant la maladie de Parkinson (MP), les syndromes parkinsoniens atypiques dégénératives avec des signes de drapeaux rouges incluant la paralysie supra-nucléaire progressive (PSP), l'atrophie multi-systématisée (AMS), la démence cortico-basale (DCB) et la démence à corps de Lewy (DCL) et les syndromes parkinsoniens secondaires (SP) aux agressions du système nerveux central par les neuroleptiques, les causes vasculaires, toxiques, amyloïde [1,2]. La gravité de ces maladies est le handicap moteur sur le patient qui évolue vers un état grabataire et le décès du patient. Dans les pays en voie de développement une augmentation de l'espérance de vie a été observée augmentant ainsi l'incidence des maladies liées à l'âge telle que la maladie de Parkinson. La prévalence de celle-ci en Afrique, actuellement est estimée à 1.3 millions de patients [3]. Les données épidémiologiques et les profils cliniques sur les syndromes parkinsoniens à Madagascar ne sont pas disponibles à ce jour. Un long travail sera nécessaire pour pourvoir ces données. Notre équipe s'est d'abord intéressée sur une étude clinique dont l'objectif est de décrire la fréquence et le profil clinique des syndromes parkinsoniens dans notre Service de Neurologie en prélude d'une étude plus étendue secondairement.

## Méthodes

Nous avons mené une étude rétrospective descriptive allant de janvier 2014 à juin 2018 dans le service de Neurologie, Befelatanana, Antananarivo, le centre de référence de prise en charge de maladie neurologique à Madagascar. Nous avons collecté à partir du registre des patients vus en consultation externe, en hospitalisation de jours et en hospitalisation conventionnelle dans le service les données des patients diagnostiqués comme ayant un syndrome parkinsonien. La définition de nos cas de syndrome parkinsonien est basée sur les données cliniques et les étiologies présumées. Un patient est considéré comme ayant un syndrome parkinsoniens s'il présent les signes cardinaux à savoir les des tremblements, la rigidité, la bradykinésie, les troubles de la posture ou de la marche. La maladie de parkinson idiopathique était est diagnostiquée si le patient a les signes cardinaux sans étiologie secondaire identifiable cliniquement et sans les signes de drapeaux rouges à savoir un début symétrique, déficit sensitivo-moteur, relation temporel entre la prise de toxique ou de médicament, parkinsonisme des membres inférieurs, syndrome pyramidale, un trouble oculomoteur, une dysautonomie, non réponse au test dopaminergique [2]. Le diagnostic de parkinsonisme secondaire et atypique était basé sur la constellation d'étiologie secondaire [4,5]. Nous avons divisé notre population d'étude en patients ayant une MP et patients ayant un autre syndrome parkinsonien. Les autres syndromes parkinsoniens regroupent les SP atypiques avec les signes de drapeaux rouges ainsi que les SP secondaire à une lésion cérébrale. Nous avons collecté les données démographiques dont l'âge de début (un patient jeune est inférieur à 50 ans), l'âge de diagnostic, le délai diagnostique, le genre (masculin ou féminin); les données cliniques comme les antécédents familiaux de syndrome parkinsonien, le symptôme initial (tremblements de repos, troubles de la marche, rigidité, lenteurs), la répartition des manifestations motrices de la maladie de Parkinson (tremblements dominants, mixte ou akineto-rigide), la sensibilité du syndrome parkinsonien lors du test à la dopamine, les résultats du test cognitif MMSE (Mini-Mental Status Evaluation). La sévérité de la maladie était mesurée par le score de Hoëhn et Yahr avec le score Unified Parkinson's Disease Rating Scale (UPDRS). Le traitement utilisé comme l'anticholinergique seul ou agoniste dopaminergique ou levodopa ou autres médicaments associés était aussi collecté. Pour les autres syndromes parkinsonien, le symptôme initial ou motif référence, les étiologies étaient aussi relevées. Nous en avons mesuré les fréquences et comparé les patients avec la Maladie de Parkinson idiopathique et les autres syndromes parkinsoniens. Les données étaient traitées par le logiciel R. Les variables continus étaient exprimés en médiane et moyenne avec déviation standard et extrêmes. Les moyennes étaient comparées par le t test de Student. Le test de chi carré était utilisé pour comparer les variables catégoriques présentés par proportion. La valeur de p < 0.05 était considérée comme statistiquement significative.

## Résultats

Au terme de notre étude, nous avons retenu 104 sur 3528 patients vus dans notre service avec un diagnostic de syndrome parkinsonien. Parmi la population d'étude, 67 (64,42%) avaient une maladie de Parkinson idiopathique (MP) et 37 cas (35,47%) présentaient un syndrome parkinsonien secondaire ou atypique. L'intervalle moyen entre le début de la maladie et la prise en charge dans le service était de 2,5 ans (Tableau 1, Tableau 2). Pour les Maladies de Parkinson idiopathiques, l'âge moyen de début était à 58,5 [23; 80] ans, l'âge du diagnostic à 62 [28; 83] ans et 35 (52,23%) étaient des femmes. seulement 3 (4,47%) avaient un antécédent familial de syndrome parkinsonien. Les patients parkinsoniens idiopathiques avec tremblements de repos étaient de 65,67%, leur médiane de score de Hoehn et Yahr était de 2.0. Les formes de MP étaient tremblantes dans 24 (35,42%), mixte 28 (41,71%) et akineto-rigide 15 (22,38%) des cas. Leur UPDRS à 26 et leur MMSE était à 29 [20; 30]. Les MP étaient plus sensible au DOPAMINE (p=0,006) et leur traitement était le L-Dopa dans 61,19% des cas (Tableau 2, Tableau 3) Pour les autres syndromes parkinsoniens, 27 (72,97%) étaient des hommes, l'âge moyen de début était à 57,5 [26; 83] ans, l'âge du diagnostic à 59,3 [26; 83] ans avec 2 (5,40%) patients ayant un antécédent familial de syndrome parkinsonien. Les tremblements étaient présents dans 35,13%. Les étiologies étaient dominées par l'atrophie multi-systématisée avec 17/37 (46,64%). Les patients autres ayant un syndrome parkinsonien avaient plus de troubles cognitifs (p=0,0306) avec un MMSE à 27,48 [11; 30] (Tableau 3, Tableau 4).

**Tableau 1:** fréquences des syndromes parkinsoniens vus dans le Service de Neurologie, Hôpital Befelatanana, Antananarivo

	Fréquence (N)	Pourcentage (%)
**Fréquence absolue**	104/3528	2,94
**Fréquence relative**		
**Genre**		
Masculin	**60/1842**	**3,25**
Féminin	44/1686	2,60
**Syndrome parkinsonien**		
Idiopathique	**67/104**	**64,42**
Secondaire et atypique	37/10424	35,47

**Tableau 2:** caractéristiques des syndromes parkinsoniens vus dans le Service de Neurologie, Befelatanana, Antananarivo

variables	Idiopathique (N =67)	Secondaire et atypique (N = 37)	p
**Age**			
De début	58,5 [23; 80]	57,5 [26; 83]	0,613
De diagnostic	62 [28; 83]	59,3 [26; 83]	0,281
**Délai diagnostique**	3	2	0,106
**Genre**			
Masculin	32 (47,76%)	27 (72,97%)	5
Féminin	35 (52,23%)	10 (27,02%)	
**Antécédents**			
Sans particularité	40 (59,70%)	21 (56,75%)	0,085
Familial de SP*	3 (4,47%)	2 (5,40%)	0,832
MMSE**	**29 [20; 30]**	**27,48 [11; 30]**	**0,0306**
**Sensibilité dopamine**			
Oui	**61 (91,04%)**	**26 (70,27%)**	**0,006**
Non	6 (8,95%)	11 (29,72%)	0,613

* **SP**: Syndrome Parkinsonien

** **MMSE**: Mini Mental Status Evaluation

**Tableau 3:** caractéristiques propres des maladies de Parkinson vus dans le Service de Neurologie, Befelatanana, Antananrivo

Variables	Nombre	Pourcentage (%)
**Symptômes initials**		
Tremblements de repos	**44**	**65,67**
Troubles de la marche	9	13,43
Rigidité	9	13,43
Lenteur moteur	5	7,46
**Types**		
Tremblements dominants	24	35,42
Mixte	**28**	**41,71**
Akineto-rigide	15	22,38
**Jeune**	15	22,38
**Autres**		
Test MODOPAR: Positif	**60**	**89,55**
Score Hoehn et Yahr (médiane)	**2**	
UPDRS*	**26**	
**Médicaments**		
Anticholinergique seul	3	4,47
Agoniste dopaminergique	22	32,83
L-Dopa	**41**	**61,19**
Autres	3	4,47

* **UPDRS**: United Kingdom Parkinson Disease Rating Scale

**Tableau 4:** caractéristiques des parkinsonismes secondaires et atypiques vus dans le Service de Neurologie, Befelatanana, Antananarivo

Variables	Nombre	Pourcentage (%)
**Symptômes initials ou références**		
Troubles neurologiques	1	2,70
Tremblements	13	35,13
Troubles de la marche	11	29,79
Syndrome parkinsonien	3	2,10
Lenteur moteur	5	13,51
Rigidité	4	10,81
**Etiologie**		
**Atypique**		
Atrophie Multi-Systémasée	17	46,64
Paralysie Supra-nucléaire Progressive	2	5,40
Démence Cortico-Basale	2	5,40
Démence à Corps de Lewy	4	8,80
Secondaire	11	29,72

## Discussion

Malgré ses les imperfections méthodologiques de notre étude (car elle est monocentrique en intra-hospitaliers avec un faible nombre d'échantillon et rétrospective), elle est la première à Madagascar sur les syndromes parkinsoniens permettant d'étayer les caractéristiques des patients atteints de maladie de parkinson et de syndrome parkinsoniens secondaires ou atypique dans un centre de référence national. Et pour chaque patient avec MP des scores UPDRS et Hoëhn et Yahr ont pu être établi. En effet, nous avons pu décrire sur le plan clinique 104 patients présentant un syndrome parkinsoniens sur 3528 patients vus dans notre service soit 2,94%. La majorité de cas dans cette population d'étude avaient une maladie de Parkinson idiopathique (MP) tandis que les syndromes parkinsoniens secondaires et atypiques ne touchaient qu'environ un quart des cas. En 1991, en Europe en population générale 2-3% avait un parkinsonisme. La prévalence du parkinsonisme augmente avec l´âge [6]. Dans l'étude d'Okobadejo *et al.* La fréquence intra-hospitalière du parkinsonisme était de 1.47%. Avec 79% de MP et 21 % de parkinsonisme secondaire [2]. Cela pourrait s'expliquer par le fait que nous avons eu une période d'étude plus courte par rapport à Okobadejo. Chez les Nord-américains en 1972, ils avaient une incidence annuelle de 20,5/100,000 avec à peu près la même prévalence: 300/100,000 individus. L'incidence augmentait avec l'âge [1]. Dans notre étude, l'âge moyen de début de leur syndrome était de 58 ans. L'intervalle moyen entre le début de la maladie et la prise en charge dans le service était de 2,5 ans. Dans une étude d’El-Tallawy *et al.* sur 15,482 sujets avec une prédominance masculine, un âge moyen de 40 ans. Quarante-neuf personnes avaient un parkinsonisme, donnant une prévalence de 0,316/100 (95% CI 240.2-404.9) [7]. Cela pourrait être dû à leur très grand nombre de population et leur âge moyenne plus jeune car la prévalence augmente avec l'âge mais devenant plus faible chez les personnes très âgées [8]. Dans une autre étude, leur âge de début de la maladie était à 48 ans mais leur étude était faite il y a plus de 40 ans [1].

Selon McInerney-Leo A *et al.* en 2004, il existe moins de parkinsonisme chez les personnes de races noires que chez les blancs avec une prévalence entre 4,6 et 4,3. L'âge moyen était plus jeune chez les noirs [9]. Mais actuellement dans les pays en voie de développement une augmentation de l'espérance de vie a été observée ainsi une augmentation des maladies reliées à l'âge telle que la maladie de Parkinson dans ce sens. Le nombre de celle-ci en Afrique est estimé à 1,3 millions de patients d'après Cilia R *et al.* en 2011 [3]. Pour les Maladie de Parkinson l'âge moyen de début était à 58,5 ans [23; 80] et l'âge du diagnostic à 62 ans [28; 83]. Trente-cinq (52,23%) étaient des femmes. Ils avaient un score UPDRS à 26. La médiane de score de Hoehn et Yahr était de 2. Les formes de MP étaient tremblantes dans 24 (35,42%), mixte 28(41,71%) et akineto-rigide 15 (22,38%) des cas. Une étude en Egypte en 2013, avait un âge de début à 57,8 ans et 69,40 ans à la présentation. Il n'y avait pas de différence entre l'incidence féminine et masculine. Leur score UPDRS était de 5775 et la plupart avaient un score de H et Y à 2. La forme de MP tremblants dominants étaient de 51,50%, mixte 27,30% et 21,20% akinéto-rigide [7]. Dans une autre étude au Nigeria, un âge moyen de début de 61.5 ans, une prédominance masculine, un UPDRS à 57,7 et une médiane de H et Y à 2 étaient observés. Les MP étaient tremblement dominant dans 31,6%, mixte dans 55,1% et akinéto-rigide dans 14,3% [2]. Leur UPDRS élevé peut être dû au délai diagnostique plus long que le nôtre. Wong *et al.* ont noté un âge de début de 64.4 ans avec un retard de diagnostic de 1,9 ans. Pour les MP jeune, le délai est de 7 ans. Donc, un jeune âge au début est associé à un délai plus long pour le diagnostic [8]. Dans d'autres études antérieures, l'incidence de la MP augmentait rapidement après l'âge de 60 ans. Le taux chez l'homme était 91% plus que chez les femmes, sauf au niveau du groupe asiatique [10]. Seulement 11% de la population avaient un âge de plus de 50 ans [11]. Vers la fin de l'année 2015, 166 712 personnes ont été traitées pour MP en France, soit une prévalence de 2,50 patients pour 1 000 personnes. Au cours de cette même année, le nombre de personnes nouvellement traitées étaient 25 842, soit une incidence de 0,39 patients pour 1 000 personnes-années. La prévalence et l'incidence augmentaient progressivement avec l'âge jusqu'à 80 ans; plus de la moitié des patients avaient plus de 75 ans. Les hommes étaient atteints environ 1,5 fois plus souvent que les femmes [12]. La prévalence de la MPI est plus élevée chez les personnes âgées mais faible chez les personnes jeunes. Il existe une prédominance masculine, car il y a le rôle protecteur probable de l'œstrogène chez la femme [13-15]. La prédominance féminine dans notre étude pourrait s'expliquer par notre faible nombre d'échantillon.

McInerney-Leo A *et al.* ont trouvé que 0,15% des noirs ont une MP contre 2,3% des blancs [9]. En Afrique les MPI augmente mais les données sont limitées. Plus de 50% sont non diagnostiqués si on prend en compte les pays en développement à cause de la non connaissance des symptômes et des signes cliniques. Tout est mise sur le compte de l'avancé en âge des patients pas par la MPI, cependant les noirs étaient plus jeunes [10,16]. Pour les parkinsoniens-secondaire et atypique, vingt-sept soit 72,97% étaient des hommes. L'âge moyen de début était à 57,5 [26; 83] ans et l'âge du diagnostic à 59,3 [26; 83] ans. Les étiologies étaient dominées principalement par l'atrophie multi-systématisée avec 17/37 (46,64%). Les autres étiologies étaient surtout dominées par les syndromes parkinsoniens dues aux neuroleptiques. Dans une étude d'Okobadejo *et al.* un âge moyen de début de 57,5 ans avec une prédominance masculine était observé. Leurs étiologies étaient dominées par le parkinsonisme vasculaire dans 34,6%, un parkinsonisme par médicaments dans 19,2 % et AMS dans 15,4% [2]. Dans une autre étude 33,6% des patients avaient un parkinsonisme secondaire dont 28,6% d'origine vasculaire [7]. La fréquence élevée des AMS dans notre étude pourrait faire l'objet d'étude ultérieure. Le Parkinsonisme secondaire compte 0.5% de noirs contre 1.15% de blancs [9]. Actuellement, l'incidence en Afrique tend à augmenter car la population augmente en âge [10]. Les patients parkinsoniens secondaire avaient plus de troubles cognitifs (p=0,0306) et les MP étaient plus sensible au DOPAMINE (p=0,006). Dans les formes tremblements dominantes des MPI, nous avons associés le L dopa avec de l'anticholinergique mais ce dernier pourra aggraver les troubles cognitifs, ces troubles peuvent aussi être associés à l'avancé de la maladie [17]. Tandis que dans les parkinsonismes secondaires, ces troubles font parties des signes de drapeaux rouges si ils surviennent précocement [1,2]. Les troubles cognitifs étaient à 24,20% dans une étude d’El-Tallawy HN*et al.* [7]. Nos patients avec MPI ont reçu du Levodopa dans 61,19% et des agonistes dopaminergiques dans 32,83 des cas. Dans une étude au Nigeria, 100% de la population d'étude étaient sous L dopa et 75/98 étaient suivi. Vingt-huit sur vingt-neuf étaient sous dopa agoniste, bromocriptine et 1 MP sous Ropirinole [2]. Même diagnostiqué les personnes avec MP ont un problème avec la disponibilité, le moyen de s'offrir et l'assurance de l'existence des médicaments au long terme [16]. Dans les pays en développement les MP ne sont pas assez diagnostiquées et pas assez traitées à cause de non disponibilité de spécialiste et pas assez de médicaments, ni de suivi, ni d'éducation. Actuellement, approximativement 86% de la population vient dans ces pays en développement et les aides internationaux sont focalisés à sur la malnutrition, et les pathologies infectieuses [3]. Ainsi, les pathologies dégénératives ne sont pas en priorité [10]. Cependant, il a été démontré que l'utilisation du Levodopa et l'amélioration de système de santé augmente la longévité [1].

## Conclusion

Les syndromes parkinsoniens ne sont finalement pas aussi rares à Madagascar, mais peuvent être sous-diagnostiqués. La maladie de Parkinson idiopathique domine largement en termes de fréquence. Le diagnostic est relativement aisé pour la maladie de Parkinson alors que les autres syndromes parkinsoniens nécessitent plus souvent le recours aux examens d'imagerie cérébrale peu accessible et peu disponible dans le pays. La majorité des patients sont vus dans le service de référence avec un retard de quelques années comme dans les autres pays en voie de développement. Il existe aussi le problème de non accessibilité au traitement des syndromes parkinsoniens, il faut aussi attirer les aides internationaux sur ces pathologies. La méconnaissance de la maladie par la population et le manque d'orientation des patients par les personnels de santé aux spécialistes seront les gaps à améliorer afin de répertorier et de prendre en charge les syndromes parkinsoniens à Madagascar.

### État des connaissances actuelles sur le sujet

- Les caractéristiques cliniques des syndromes parkinsoniens dans d'autres pays africains.

### Contribution de notre étude à la connaissance

- Les caractéristiques cliniques des syndromes parkinsoniens à Madagascar;
- L'utilisation de différents scores de sévérité de la Maladie de Parkinson.

## Conflits d’intérêts

Les auteurs ne déclarent aucun conflit d’intérêts.
